# Prevalence and risk factors for newborn anemia in southwestern Uganda: a prospective cohort study

**DOI:** 10.21203/rs.3.rs-3054549/v1

**Published:** 2023-06-26

**Authors:** Joseph Ngonzi, Leevan Tibaijuka, Timothy Mwanje Kintu, Raymond Bernard Kihumuro, Ahabwe Onesmus, Byamukama Onesmus, Julian Adong, Wasswa Salongo, Adeline A. Boatin, Lisa M. Bebell

**Affiliations:** Mbarara University of Science and Technology; Mbarara University of Science and Technology; Mbarara University of Science and Technology; Mbarara University of Science and Technology; Mbarara University of Science and Technology; Mbarara University of Science and Technology; Mbarara University of Science and Technology; Mbarara University of Science and Technology; Massachusetts General Hospital; Harvard Medical School, Massachusetts General Hospital

**Keywords:** Maternal anemia, newborn anemia, cord blood, risk factors, prevalence, Uganda, prospective cohort

## Abstract

**Introduction:**

The global prevalence of anemia in pregnancy is about 42%, and in sub-Saharan Africa, the prevalence of newborn anemia ranges from 25–30%. Anemia in newborn babies may cause complications such as delayed brain maturation and arrested growth. However, there is limited data on prevalence of newborn anemia and its risk factors in people living in resource-limited settings.

**Objectives:**

We determined the prevalence and risk factors for newborn anemia and its correlation with maternal anemia in southwestern Uganda.

**Methods:**

This was a prospective cohort study of 352 pregnant women presenting to Mbarara Regional Referral Hospital for delivery. We collected maternal blood in labor and umbilical cord blood from the placental vein, as a proxy for newborn hemoglobin. We estimated hemoglobin using a point-of-care Hemocue machine. We used summary statistics to characterize the cohort, and compared demographic characteristics and outcomes using Chi-square, t-test, and Wilcoxon Ranksum analyses. We defined newborn anemia as umbilical cord hemoglobin < 13g/dl and estimated the relationship between maternal and umbilical cord hemoglobin using linear regression analysis, adjusting for potential confounders.

**Results:**

The prevalence of newborn anemia was 17%. The average maternal parity was significantly higher for anemic and non-anemic newborns (3.5 versus 2.8, *P = 0.01*). Mean age [SD] was significantly lower for participants with umbilical cord hemoglobin < 13g/dl than those > = 13 g/dl, (26 [5.6] versus 28 [6.3], *P* = 0.01). In multivariable linear regression analysis, a 1-point decrease in maternal hemoglobin was associated with a 0.14-point decrease in umbilical cord hemoglobin (*P* = 0.02). Each one-unit increase in maternal parity was associated with a 0.25-point decrease in umbilical cord hemoglobin (*P* = 0.01). Cesarean delivery was associated with a 0.46-point lower umbilical cord hemoglobin level compared to vaginal delivery (*P* = 0.03).

**Conclusions:**

We found a significant correlation between maternal and newborn hemoglobin levels, underscoring the importance of preventing and correcting maternal anemia in pregnancy. Furthermore, maternal anemia should be considered a risk factor neonatal anemia.

## Background

Anemia in pregnancy is defined as a Hemoglobin (Hb) level <11g/dl (World Health Organization, 2015). The global prevalence of anemia in pregnancy is approximately 42% (Garzon et al., 2020). In Uganda, the prevalence has been estimated at 22% - 33% (Ononge, Campbell and Mirembe, 2014; Obai, Odongo and Wanyama, 2016), though a recent study in Uganda reported a very high prevalence of 63% among pregnant women attending antenatal care at Mbarara Regional Referral Hospital (Okia et al., 2019). Maternal anemia can be due to blood loss, infection, and other causes, but nutritional deficiency is the most common cause globally (Zainel et al., 2018). Inadequate iron intake and poor infrastructure services for early diagnosis and treatment of anemia at the health facility level in resource limited settings (RLS) (Carter et al., 2010) contribute to anemia, and pregnant women are one of the most vulnerable groups (Garzon et al., 2020). Iron needs increase exponentially during pregnancy to meet the demands of the feto-placental unit, expand maternal erythrocyte mass, and compensate for iron lost through blood during delivery (Garzon et al., 2020; Means, 2020).

Iron deficiency anemia in pregnancy may also affect the hemoglobin and iron reserves in offspring, leading to newborn anemia (Shukla, Srivastava and Verma, 2019; James, 2021). Anemia in newborns can be life-threatening (O. and O., 2012; Kassebaum et al., 2014), causing delays in brain maturation, tissue hypoxia, arrested growth, and poorer cognitive, motor, and social-emotional development (Hawsawi et al., 2015; Mhanna et al., 2016; Zhang et al., 2016; Moniruzzaman Mollah et al., 2021). The proportion of newborn infants with anemia in sub-Saharan Africa ranges from 23% to 66% (Brabin et al., 2004; Dairo and Lawoyin, 2004; Tiruneh, Shiferaw and Enawgaw, 2020). One study found an overall positive correlation between maternal and umbilical cord hemoglobin (as a marker of newborn anemia), with lower umbilical cord blood hemoglobin levels related to anemic mothers (Zainel et al., 2018).

However, there is a paucity of data on the strength of the correlation between maternal and newborn anemia, overall prevalence, and maternal risk factors for neonatal anemia in RLS. Such data are urgently needed to institute early interventions to reduce anemia-associated complications. Specifically, in Uganda, despite a number of published studies on prevalence of maternal anemia, there is a dearth of information regarding the prevalence and risk factors for newborn anemia. To address this knowledge gap and contribute to development of public health guidelines, we designed a prospective cohort sub-study to assess the prevalence, correlation between cord blood and maternal anemia and risk factors for newborn anemia in southwestern Uganda.

## Methods

### Participant recruitment and ethics:

This was a prospective cohort study of 352 pregnant women presenting to Mbarara Regional Referral Hospital (MRRH) for delivery. The hospital has a total bed capacity of 420 beds and serves largely a semi-urban population. The study population consisted of 176 women living with HIV (WLWH) and 176 HIV-uninfected women and the babies born to them. All women ≥18 years of age presenting in labor for delivery were eligible for enrolment. Women were excluded from the study if they did not speak English or Runyankole (the local language) well enough to give informed consent, had known or suspected multiple gestation, could not be reached by telephone after discharge for follow-up, were WLWH but not taking antiretroviral therapy (ART), or the study team was unable to collect the participant’s placenta. All participants included in the parent study were included in this preplanned anemia analysis.

### Ethical approval:

The study was approved by the institutional ethics review board at Mbarara University of Science and Technology (MUST, 11/03–17), Partners Healthcare (2017P001319/MGH), and the Uganda National Council of Science and Technology (HS/2255). Written informed consent was obtained from all the participants, including consent to review the medical records of the woman and her baby for clinical history, treatment received, and outcome measures.

### Sample collection and hemoglobin determination:

After written informed consent was obtained, maternal blood was collected via peripheral venipuncture. At delivery, the placenta was collected, and umbilical cord blood was obtained via venipuncture of the umbilical vein within 30 minutes of placental delivery. One drop of blood was used for hemoglobin determination using the point-of-care HemoCue Hb 301 according to the manufacturer’s instructions (HemoCue, USA). Umbilical cord hemoglobin (Hb) was used as a proxy for newborn hemoglobin and newborns were classified as having polycythemia (Hb>20g/dl), normal (13–20g/dl) and anemia (<13g/dl). The primary outcome variable was umbilical cord hemoglobin level less than 13g/dl. Maternal anemia was defined as Hb <11g/dl (Ononge, Campbell and Mirembe, 2014; WHO, 2015).

### Sample size and statistical analysis:

The sample size of 352 participants was selected to achieve sufficient power for the primary outcome, comparing the prevalence of placental inflammation by maternal HIV serostatus. Summary statistics were used to characterize the cohort. Demographic characteristics and outcomes were compared between umbilical cord blood hemoglobin below 13g/dl and 13g/dl or more using Chi-squared analysis for categorical variables and student’s t-test or Wilcoxon Ranksum for continuous variables. Variables with *P*-values < 0.2 in association with newborn anemia were selected for the multivariable analysis. We then estimated the relationship between maternal and umbilical cord hemoglobin concentrations using linear regression analysis, adjusting for potential confounders. Predictor variables were selected using the P-value screen on bivariate analysis as above and based on published associations, including maternal age, parity, employment, maternal HIV serostatus, maternal self-reported malaria diagnosis in pregnancy, mode of delivery, number of antenatal care visits, maternal report of deworming during pregnancy, maternal report of anemia diagnosed during pregnancy, diagnosis of antepartum hemorrhage (APH), maternal report of intake of ferrous iron/folic acid intake during pregnancy and educational status. All variables with *P*-values < 0.05 in the final multivariate linear regression model were considered significant independent predictors of the outcome of newborn anemia. All analyses were performed using Stata software (Version 16.0, StataCorp, College Station, TX).

## Results

Of 352 maternal participants recruited, 60/352 had umbilical cord hemoglobin levels < 13g/dl, representing a prevalence of newborn anemia of 17.0% (Table 1). Of the 352 participants, 281 (79.8%) had normal hemoglobin level while 11 (3.2%) had polycythemia. Mean age (SD) was significantly lower for participants with umbilical cord hemoglobin <13g/dl than those >=13 g/dl, (26 [5.6] versus 28 [6.3], *P*=0.01). Mean birthweight did not differ significantly between participants with umbilical cord hemoglobin < 13g/dl and those with >= 13 g/dl (3.1 versus 3.2 kilograms, *P*=0.95, Table 1). There was no significant difference between the two groups in terms of maternal and newborn profiles except that average parity for the anemic participants was 3.5, significantly higher than non-anemic participants 2.8 (*P*=0.01, Table 1).

**Figure 1:**
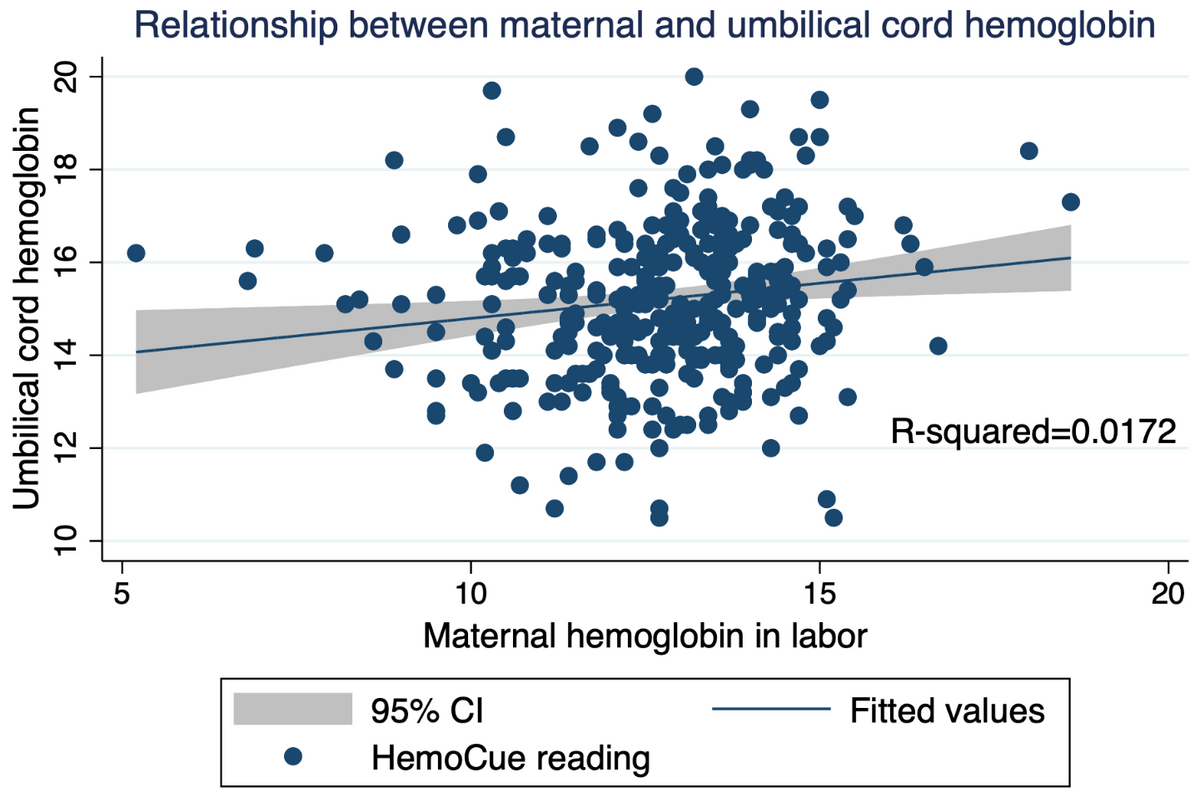
Scatter plot showing mean hemoglobin concentration of maternal and umbilical cord blood

There was a significant positive correlation between maternal and umbilical cord haemoglobin concentrations (Figure 1, *R*^*2*^ = 0.017).

In multivariable linear regression analysis, for every one-point increase in maternal hemoglobin, there was a corresponding 0.15 point (unadjusted) / 0.14 point (adjusted) increase in umbilical cord hemoglobin and the result was statistically significant (*P*=0.02, Table 2). For every one-unit increase in maternal parity, there was a 0.15 point (unadjusted) / 0.25 point (adjusted) decrease in umbilical cord hemoglobin and the association was statistically significant (*P*=<0.01). Cesarean delivery mode was also associated with a 0.39 point (unadjusted) / 0.46 point (adjusted) decrease in umbilical cord hemoglobin level (*P*=0.03, Table 2). The lowest asset index quartile was significantly associated with decreased hemoglobin (*P*=0.03), indicating that lower socioeconomic status was associated with anemia. Maternal report of receiving iron and/or folate in pregnancy, de-worming in pregnancy, diagnosis of malaria in pregnancy, and number of antenatal care visits during pregnancy were not significantly associated with anemia in adjusted or unadjusted analyses.

## Discussion

This study aimed to assess the prevalence of anemia, association between umbilical cord anemia and maternal anemia, and risk factors for newborn anemia at a regional referral hospital in southwestern Uganda.

The prevalence of newborn anemia in this study was 17%, lower than the prevalence reported in previous cross-sectional studies. For example, prior studies in Ethiopia reported a prevalence of newborn anemia ranging from 23% to 25% (Tiruneh, Shiferaw and Enawgaw, 2020; Alamneh et al., 2022). However, these studies were conducted in a largely rural setting compared to our semi-urban study population. Prior studies in RLS have noted that anemia is more prevalent in rural than urban and semi-urban populations (Tesfaye, Tessema and Jarso, 2020), which may partially explain these differing findings. In contrast, the prevalence of anemia in our populations was higher than what has been reported in the USA, Nepal and Ethiopia which reported prevalence rates of 14%, 6% and 9%, respectively (Terefe et al., 2015; Lee et al., 2016; Timilsina et al., 2018). These differences in anemia prevalence could be attributed to differences in socioeconomic conditions and clinical characteristics of the study participants. Additionally, the differences could also be because in some previous studies, Specifically (Terefe et al., 2015), women with only iron deficiency anemia (IDA) were included, whereas our study included anemia of any cause.

We found a significant positive correlation between maternal and umbilical cord hemoglobin concentration. For every 1-point increase in maternal hemoglobin, there was a corresponding 0.14-point increase in umbilical cord hemoglobin. The finding of a positive correlation between maternal and umbilical cord haemoglobin in this study is similar to other studies in Nigeria, Kenya, Israel, India and Iran (Kuile et al., 1999; Dapper and Didia, 2007; Alizadeh et al., 2014). Since the fetus obtains iron from maternal transferrin, when maternal iron stores are depleted, the fetus cannot accumulate as much iron resulting in a decrease in fetal iron stores and reduced fetal hemoglobin levels (Bergmann et al., 2010; Kosto et al., 2015; Shukla, Srivastava and Verma, 2019; Alegbeleye, Abam and Orazulike, 2020; Rathoria and Rathoria, 2021). The physiological changes and metabolic demands of pregnancy result in an increased requirement for iron in pregnancy (Kohli, Rajput and Venkatesan, 2021) predisposing to maternal anemia. Maternal anemia leads to adaptations in placental and fetal physiology resulting in pregnancy and birth complications such as low birth weight, neurodevelopmental disorders and premature delivery (Dane et al., 2013; Wiegersma et al., 2019; Rahmati et al., 2020).

We also found a statistically significant association between parity and umbilical cord hemoglobin levels, with a 0.25-point decrease in umbilical cord hemoglobin for every one-unit increase in parity (*P<0.01*). Higher parity increases the risk of developing iron deficiency anemia in pregnancy, and the incidence of anemia has been shown to increase with the number of pregnancies (Kumari and Badrinath, 2002; Çelik Kavak and Kavak, 2017; Shah, Warsi and Laghari, 2020; Vionalita and Permata, 2020). This association could be attributed to depletion of maternal iron stores with each subsequent pregnancy or to inadequate spacing between pregnancies that often comes with high parity and is common among Ugandan mothers (McGuire and Stephenson, 2015; Aleni, Mbalinda and Muhindo, 2020; Tiwari and Mishra, 2020; Belaid et al., 2021).

In addition, we found that cesarean delivery was significantly associated with a 0.46-point decrease in umbilical cord hemoglobin level. Cesarean delivery has previously been described to increase the risk of postpartum anemia by two-fold (Bergmann et al., 2010), due to the increased risk of uterine atony and severed vessels when the abdominal wall is opened (Joseph et al., 2007; Kramer et al., 2013). Lastly, we found that lower wealth, measured using the asset index, was associated with lower umbilical cord hemoglobin levels. These findings are similar to those in other settings (Lokare et al., 2012; Hooja et al., 2020), although a study in Indonesia found no direct relationship between the socioeconomic status and anemia (Faculty of Medicine, Universitas Methodist Indonesia et al., 2019). Women with lower socioeconomic status may have limited access to nutritious food, which can lead to poor maternal nutritional status and subsequent fetal anemia (Pell et al., 2013). We found a significant positive correlation between maternal and newborn hemoglobin levels. Parity, cesarean delivery, and lower asset index quartile were significantly associated with newborn anemia. Such information is required to institute early interventions to reduce anemia-associated complications. This underscores the importance of preventing maternal anemia and maintaining adequate iron stores in pregnancy.

## Conclusion

The prevalence of newborn anemia in this study was 17%. We found a significant positive correlation between maternal and umbilical cord hemoglobin concentration. We also found a statistically significant association between parity and umbilical cord hemoglobin levels, with a 0.25-point decrease in umbilical cord hemoglobin for every one-unit increase in parity. Cesarean delivery was significantly associated with a 0.46-point decrease in umbilical cord hemoglobin level.

Based on our results, we recommend that obstetric care policy guidelines need to incorporate routine monitoring of maternal hemoglobin levels. In addition, umbilical cord hemoglobin may also be useful for early diagnosis and intervention for newborn anemia. Efforts should also be made to improve the socioeconomic status of pregnant women and provide them with adequate access to antenatal care and nutritional support. Overall, ensuring adequate iron stores during pregnancy, through good nutrition and iron supplementation and spacing pregnancies appropriately are key policy strategies for preventing maternal anemia and its associated complications in sub-Saharan Africa and other RLS.

## Figures and Tables

**Table 1:** Sociodemographic characteristics and clinical data of study participants, compared by umbilical cord hemoglobin level

Maternal and newborn characteristics	Umbilical cord blood Hb >= 13 g/dl	Umbilical cord blood Hb <13g/dl	P-value
Resides in Mbarara, n (%)	204 (64.2)	23 (70.0)	0.81
Formally employed, n (%)	118 (37.0)	9 (27.3)	0.27
Delivered by Cesarean, n (%)	101 (32.4)	13 (39.4)	0.37
Married, n (%)	30 (90.1)	287 (90.0)	0.86
Primary education or less, n (%)	185 (58.8)	22 (58.0)	0.34
Attended ≥ 4 ANC visits, n (%)	200 (62.7)	23 (69.7)	0.43
Maternal self-report of no anemia diagnosed during pregnancy, n (%)	315 (98.8)	33 (100.0)	0.52
Maternal self-report of deworming during pregnancy, n (%)	273 (85.6)	28 (84.8)	0.91
Maternal self-report of iron/folate intake during pregnancy, n (%)	219 (68.7)	23 (69.7)	0.90
Maternal self-reported malaria diagnosis in pregnancy, n (%)	38 (11.9	4 (12.1)	0.97
HIV-infected, n (%)	155 (48.6)	21 (63.6)	0.10
Parity, mean (SD)	2.8 (1.6)	3.5 (1.6)	0.01
Age, mean (SD)	26 (5.6)	28 (6.3)	0.01
Birthweight, mean (SD)	3.2 (0.4)	3.1 (0.5)	0.95

Footnotes: Hb – Hemoglobin; HIV – Human Immunodeficiency Virus; ANC – Antenatal Care; SD – Standard Deviation

**Table 2:** Univariable and multivariable linear regression analysis of risk factors for newborn anemia

	Univariable		Multivariable	
Characteristic	b coefficient (95% C)	P-value	b coefficient (95% C)	P-value
Maternal hemoglobin	0.15 (0.04 – 0.27)	0.01	0.14 (0.03 – 0.26)	**0.02**
Age	−0.03 (−0.06 – 0.01)	0.14	0.03 (−0.02 – 0.09)	0.22
Parity	−0.15 (−0.27 – 0.04)	0.01	−0.25 (−0.43 – −0.07)	**<0.01**
Cesarean delivery	−0.39 (−0.81 – 0.26)	0.07	−0.46 (−0.88 – −0.04)	**0.03**
Self-reported intake of iron/folate in pregnancy	−0.39 (−2.26 – 1.47)	0.68	−0.56 (−2.4 – 1.3)	0.56
Self-reported malaria in pregnancy	0.10 (−0.51 – 0.71)	0.74	1.03 (0.56, 1.88)	0.93
Attended antenatal care < 4 times	0.24 (−0.17 – 0.65)	0.25	1.17 (0.78, 1.77)	0.45
Asset index quartile				
Poorest	−0.35 (−0.91 – 0.21)	0.22	−0.42 (−0.98 – 0.15)	0.15
Wealthier		0.09		0.06
Wealthiest	−0.49 (−1.05 – 0.69)	0.18	−0.55 (−1.11 – 0.01)	**0.03**
	−0.38 (−0.94 – 0.18)		−0.65 (−1.23 – −0.06)	
Self-report of deworming during ANC	−.21 (−0.35 – 0.77)	0.48	0.22 (−0.33 – 0.79)	0.42

Footnotes: ANC – Antenatal care; CI – Confidential interval
